# Exploring the Prevalence of Respiratory Failure in Adults Presenting With Acute Exacerbations of Chronic Obstructive Pulmonary Disease

**DOI:** 10.7759/cureus.63334

**Published:** 2024-06-27

**Authors:** Rizwan Ullah, Viraj Shetty, Aftab Ahmad, Albine Djeagou, Jubran Al Hooti, Gayatri Misra, Waqar Khan, Arshad Mehmood, Kashif Ali, Aizaz Afridi

**Affiliations:** 1 Internal Medicine, Hayatabad Medical Complex Peshawar, Peshawar, PAK; 2 Medicine, Victoria Hospital, Bengaluru, IND; 3 General Medicine, Cork University Hospital, Cork, IRL; 4 Faculty of Health Sciences, University of Buea, Buea, CMR; 5 Medicine, University College Dublin, Dublin, IRL; 6 Medicine, American University of Antigua, Antigua, USA; 7 Medicine, Hayatabad Medical Complex Peshawar, Peshawar, PAK; 8 Internal Medicine, Lady Reading Hospital Peshawar, Peshawar, PAK

**Keywords:** exploring, pakistan, peshawar, prevalence, respiratory failure, copd exacerbation

## Abstract

Introduction: Chronic obstructive pulmonary disease (COPD) poses a significant global health burden and is a leading cause of morbidity and mortality. Acute exacerbations of COPD often lead to respiratory failure, necessitating a thorough understanding of its prevalence. This study aimed to investigate the prevalence of respiratory failure among adult patients experiencing acute exacerbations of COPD.

Materials and methods: A descriptive, cross-sectional study was conducted over a span of seven months at the internal medicine department of Hayatabad Medical Complex, Peshawar. A total of 255 adult patients with acute exacerbations of COPD were included, and their demographic data, as well as arterial blood gas (ABG) analysis results, were collected. The prevalence of respiratory failure was defined by specific arterial blood gas criteria.

Results: The study revealed a notable prevalence of respiratory failure (41.18%) among COPD patients presenting with acute exacerbations. Factors such as older age and male gender were identified as being associated with a heightened risk of respiratory failure.

Conclusion: In conclusion, acute exacerbations of COPD predominantly affect middle-aged males (65.5%), with the 51-60 age group being the most impacted. Respiratory failure was present in over 41% of cases. ABG analysis indicated significant acid-base imbalances, hypoxemia, and hypercapnia, with compensatory chronic respiratory acidosis. These findings highlight the need for targeted interventions to manage and prevent COPD exacerbations, especially in middle-aged men.

## Introduction

Chronic obstructive pulmonary disease (COPD), a slowly progressive condition, is characterized by fixed or partially reversible airflow obstruction, denoted by a FEV1/FVC ratio below 70% [1]. Exacerbations of COPD involve a deviation from the patient's usual dyspnea, cough, and/or sputum production, exceeding normal day-to-day fluctuations. These episodes vary from temporary discomfort to progressive respiratory failure [2]. In 2019, COPD ranked as the third-highest contributor to global mortality, resulting in approximately 3.23 million deaths. The majority of COPD-related deaths among individuals under 70 years old occur in low- and middle-income countries (LMICs), accounting for nearly 90% of cases. Globally, COPD is recognized as the seventh most significant cause of disability-adjusted life years (DALYs), indicating its substantial impact on public health. While tobacco smoking is responsible for over 70% of COPD cases in high-income nations, in lower-middle-income countries, tobacco smoking contributes to approximately 30-40% of cases, with household air pollution emerging as a significant risk factor [3]. Respiratory failure frequently manifests in individuals experiencing acute exacerbations of COPD, with a prevalence rate of 35.51% [4]. Respiratory failure is defined by an arterial oxygen tension (PaO_2_) of less than 60 mmHg and/or an arterial carbon dioxide tension (PaCO_2_) greater than 45 mmHg [5]. Managing respiratory failure in COPD necessitates a holistic approach emphasizing the importance of evaluating the interaction between neuromuscular function and the workload of the respiratory system, with a focus on reversible factors. For severe exacerbations, it is advised to utilize pharmacological treatments like bronchodilators, corticosteroids, and antibiotics in conjunction with advanced respiratory support technologies. The goal is to alleviate the mechanical strain during breathing, identify and address triggering factors, and maintain optimal gas exchange while being cautious about high oxygen levels. Additionally, noninvasive ventilation is recommended. The possibility of long-term mechanical ventilation via nasal masks for specific COPD patients experiencing respiratory failure is also under consideration [6].

This study aims to investigate the prevalence of respiratory failure among adult patients presenting with exacerbations of COPD. The findings will provide valuable insights to healthcare professionals and relevant authorities, aiding in the development of necessary management strategies.

## Materials and methods

This descriptive, cross-sectional study was conducted in the internal medicine department of Hayatabad Medical Complex, Peshawar, over a period of seven months from October 2023 to April 2024. The objective of this study is to determine the prevalence of respiratory failure in COPD patients with acute exacerbations of COPD. A total of 255 patients experiencing exacerbations of COPD were enrolled using consecutive (non-probability) sampling. The sample size was calculated with a prevalence of respiratory failure of 35.51%, a confidence level of 95%, and a margin of error of 5.89% [4]. Exacerbation of COPD was defined as patients with a confirmed COPD diagnosis based on spirometry results (FEV1 less than 80% predicted and FEV1/FVC ratio less than 70%) who presented with resting dyspnea and increased sputum production. Patients were classified as having respiratory failure if their arterial oxygen tension (PaO_2_) was less than 60 mmHg and/or their arterial carbon dioxide tension (PaCO_2_) was greater than 45 mmHg, as determined by analyzing arterial blood samples using the hospital's blood gas analyzer machine. The study included male and female patients over the age of 30 diagnosed with COPD who presented with exacerbations. Exclusion criteria encompassed patients with COPD and acute severe asthma, bronchogenic carcinoma, cardiac arrhythmia, left ventricular failure with pulmonary edema, and pneumothorax, as well as those presenting with acute respiratory distress syndrome, pulmonary embolism, and respiratory failure associated with severe pneumonia. Approval was obtained from the ethical committee of Hayatabad Medical Complex, Peshawar, with the approval number Ethical Review (ERB/HMC 1849). Informed written consent was acquired from all patients upon admission, and their demographic information was recorded. A 1 ml blood sample was drawn from an accessible arterial site for arterial blood gas (ABG) analysis using a sterile 3 ml disposable syringe heparinized with 0.2 ml of heparin. The sample was promptly analyzed for partial pressures of oxygen and carbon dioxide. To control for confounding variables, strict adherence to exclusion criteria was maintained. Venous blood contamination was prevented by employing standard techniques, and blood samples were collected before administering oxygen. Study variables encompassed age, gender, presence of respiratory failure, partial pressure of carbon dioxide (PaCO_2_), partial pressure of oxygen (PaO_2_), bicarbonate level, and arterial blood pH. Data analysis was conducted using the latest version of the Statistical Package for Social Sciences (SPSS; IBM Corp., Armonk, NY). The mean ± SD was calculated for continuous variables such as age. Categorical variables, including gender and type 2 respiratory failure, were expressed as frequencies and percentages. Effect modifiers like age and sex were stratified in relation to type 2 respiratory failure. Post-stratification, a chi-square test was applied, with a p-value of less than 0.05, which was considered statistically significant.

## Results

A total of 255 patients were admitted to the medicine department with acute exacerbations of COPD, including 167 males (65.5%) and 88 females (34.5%). 29.42% of males and 11% of females had respiratory failure in acute exacerbations of chronic obstructive pulmonary disease. The male patients had an average age of 58 years ± 12.5 SD, while the female patients had an average age of 55.7 years ± 10.8 SD. The overall average age was 57.8 years ± 11.82 SD. Respiratory failure was prevalent in 105 patients (41.18%) (Tables 1-2).

**Table 1:** Gender wise frequency and percentages of respiratory failure in acute exacerbation of chronic obstructive pulmonary disease

Gender	Frequency	Percentage (%)
Male	75	29.42
Female	30	11.76
Total	105	41.18

**Table 2:** Frequency and percentages on the basis of respiratory failure

Respiratory failure	Frequency	Percentage (%)
Yes	105	41.18
No	155	58.82
Total	255	100

The most affected age group was 51-60 years, with 41 patients (16.08%), whereas the least affected age group was 30-40 years, with eight patients (3.14%) (Table 3).

**Table 3:** Frequency and percentages of patients affected with respiratory failure on the basis of age group

Age in years	Respiratory failure patients	Percentages (%)
30-40	8	3.14%
41-50	23	9.02%
51-60	41	16.08%
60>	33	12.94%
Total	105	41. 18%

Stratification of respiratory failure with respect to age group and gender with p-values of 0.039 and 0.035, which showed significant association with age groups and gender (Table 4).

**Table 4:** Stratification of respiratory failure with respect to age groups and gender

	Respiratory failure			
	Yes	No	Total	P-value
Age group				
30–40	8 (3.18%)	12 (4.7%)	20	0.039
41–50	23 (9.01%)	24 (9.41%)	47	
51–60	41(16.07%)	61 (23.92%)	102	
>60	33 (12.94%)	53 (20.78%)	86	
Gender				
Male	75 (29.42%)	92 (36.1%)	167	0.035
Female	30 (11.76%)	58 (22.7%)	88	
Total	105	150	255	

Arterial blood gas (ABG) analysis revealed that the mean pH levels for male and female patients were 7.3 ± 2.3 and 7.3 ± 2.5, respectively. The mean PaO_2_ level was 61.5 ± 7.4, and the mean PaCO_2_ level was 56.2 ± 8.0. The mean HCO_3_ level was 28.0 ± 2.0 (Table 5).

**Table 5:** Arterial blood gasses (ABGs) analysis on arrival

Gender	Mean ± SD			
	pH	PaCO_2_ (mmHg)	PaO_2_ (mmHg)	$\mathrm{HCO}_{3}^{-}$ (mmol/L)
Male	7.3 ± 2.3	55.1 ± 8.1	61.0 ± 7.5	27.9 ± 2.1
Female	7.3 ± 2.5	57.3 ± 7.8	62.3 ± 6.9	28.1 ±1.9
Total mean	7.3 ± 2.2	56.2 ± 8.0	61.5 ±7.4	28.0 ± 2.0

## Discussion

Respiratory failure is a frequent and severe complication of COPD, typically precipitated by factors such as pulmonary infections, tuberculosis, and COPD exacerbations. Utilizing noninvasive ventilation (NIV) as an initial treatment approach, with a transition to invasive ventilation when required, has proven effective in managing exacerbations of COPD and respiratory failure [7].

The objective of our study was to determine the prevalence of respiratory failure in patients presenting with acute exacerbations of COPD. Our findings indicated a 41.18% prevalence of respiratory failure in patients admitted to our department. In comparison, a previous study conducted in this region in 2010 reported a respiratory failure rate of 35.51% [4]. This increase in prevalence may be attributed to the impact of COVID-19 on respiratory mechanics, as supported by various studies [8-11]. A multicenter retrospective study conducted in four hospitals in China found that 46.5% of patients experienced respiratory failure during acute exacerbations of COPD based on arterial blood gas analysis [12].

Our study found a predominance of males affected (29.41%), which aligns with other research findings [6,7,4]. Studies suggest that the severity of COPD tends to be higher in males, especially in advanced stages. Contributing factors may include obesity and higher smoking rates, which are associated with decreased lung function and a faster decline in men. Additionally, males with COPD are more likely to experience severe pulmonary complications, such as diaphragmatic weakness, and face a higher risk of deteriorating respiratory status. These elements may explain the increased prevalence of respiratory failure among males with COPD [13].

Our study revealed that the age groups 51-60 and over 60 years were the most affected, with prevalence rates of 16.08% and 12.94%, respectively. Older age is consistently linked to a poorer prognosis in COPD. Elderly patients often have less knowledge about the disease, practice less self-care, and are more likely to be hospitalized. The complexity of COPD in older adults is exacerbated by a higher prevalence of the disease, increased symptom severity, and a greater burden of comorbidities [14,15].

Managing COPD with respiratory failure requires various interventions. Utilizing clinical pathways can significantly shorten hospital stays and enhance patient outcomes. Non-pharmacological methods like pulmonary rehabilitation and neuromuscular electrical stimulation can greatly improve quality of life. High-flow nasal cannula therapy is effective in enhancing respiratory and diaphragmatic function, alleviating dyspnea and fatigue, and balancing serum factors. Furthermore, registered nurses are essential in overseeing patients' conditions and applying suitable interventions to manage respiratory failure in COPD [16].

The limitations of this study include an increase in prevalence due to COVID-19 but do not address the specific mechanisms or quantify its impact on respiratory mechanics and COPD exacerbations. We did not divide respiratory failure into acute and chronic respiratory failure.

## Conclusions

In conclusion, respiratory failure in acute exacerbations of COPD predominantly affects males (65.5%) more than females (34.5%), with the most affected age group being 51-60 years. The average age of patients was approximately 57.8 years, highlighting that middle-aged adults are the most susceptible. Respiratory failure was a significant concern, affecting over 41% of the patients. ABG analysis showed similar pH levels for both genders, with an average of 7.3, suggesting acid-base imbalances. The mean PaO_2_ and PaCO_2_ levels of 61.5 and 56.2, respectively, indicate hypoxemia and hypercapnia, common in severe COPD exacerbations. The elevated mean HCO_3_ level of 28.0 suggests a compensatory response to chronic respiratory acidosis. Overall, these findings underscore the critical need for targeted interventions in middle-aged adults, particularly males, to manage and prevent acute exacerbations of COPD.
